# An In Situ Evaluation of Different CAM Plants as Plant Microbial Fuel Cells for Energy Recovery in the Atacama Desert

**DOI:** 10.3390/plants12234016

**Published:** 2023-11-29

**Authors:** Felipe M. Galleguillos Madrid, Mauricio Trigo, Sebastián Salazar-Avalos, Sergio Carvajal-Funes, Douglas Olivares, Carlos Portillo, Edward Fuentealba, Norman Toro, Gilda Carrasco, Luis Cáceres, Ingrid Jamett, Alvaro Soliz

**Affiliations:** 1 Centro de Desarrollo Energético de Antofagasta, Universidad de Antofagasta, Av. Universidad de Antofagasta 02800, Antofagasta 1271155, Chile; mauricio.trigo@uantof.cl (M.T.); sebastian.salazar@uantof.cl (S.S.-A.); douglas.olivares@uantof.cl (D.O.); carlos.portillo@uantof.cl (C.P.); edward.fuentealba@uantof.cl (E.F.); 2 Facultad de Ingeniería y Arquitectura, Universidad Arturo Prat, Iquique 1100000, Chile; notoro@unap.cl; 3 Department of Horticulture, Faculty of Agricultural Sciences, Universidad de Talca, Talca 3460000, Chile; gcarrasc@utalca.cl; 4 Departamento de Ingeniería Química y Procesos de Minerales, Universidad de Antofagasta, Av. Universidad de Antofagasta 02800, Antofagasta 1271155, Chile; luis.caceres@uantof.cl; 5 Centro de Economía Circular en Procesos Industriales, Universidad de Antofagasta, Av. Universidad de Antofagasta 02800, Antofagasta 1271155, Chile; ingrid.jamett@uantof.cl; 6 Departamento de Ingeniería en Metalurgia, Universidad de Atacama, Av. Copayapu 485, Copiapó 1530000, Chile; alvaro.soliz@uda.cl

**Keywords:** plant microbial fuel cell (PMFC), CAM plant, Atacama Desert, solar energy, environmental engineering, sustainability

## Abstract

Excess energy derived from photosynthesis can be used in plant microbial fuel cell (PMFC) systems as a sustainable alternative for the generation of electricity. In this study, the in situ performance of CAM (Crassulacean acid metabolism) plants in Calama, in the Atacama Desert, was evaluated for energy recovery using PMFCs with stainless steel AISI 316L and Cu as electrodes. The plant species evaluated included *Aloe perfoliata*, *Cereus jamacaru*, *Austrocylindropuntia subulata*, *Agave potatorum*, *Aloe arborescens, Malephora crocea*, and *Kalanchoe daigremontiana*. Among the plant species, *Kalanchoe daigremontiana* demonstrated significant potential as an in situ PMFC, showing a maximum cell potential of 0.248 V and a minimum of 0.139 V. In addition, the cumulative energy for recovery was about 9.4 mWh m^−2^ of the electrode. The use of CAM plants in PMFCs presents a novel approach for green energy generation, as these plants possess an inherent ability to adapt to arid environments and water-scarce areas such as the Atacama Desert climate.

## 1. Introduction

The effects of climate change, pollution, and the depletion of natural resources, with a high percentage of the population living in areas with water scarcity (over 2.4 billion inhabitants), has led to the search for new forms of sustainable energy such as photovoltaic systems [1]. Recent trends worldwide focus on developing technologies and innovations that allow for the supply of energy to the population without generating a negative impact on territories and environments. The sun is the primary source of energy that fuels most of the energy generating processes on Earth, and the sun’s energy in a year is greater than the total of all existing fossil energy resources on the planet. Despite this, there are still problems with making the most of solar resources such as (i) the discontinuity of the resource supply and (ii) the high level of radiation dispersion, which generates non-uniformity of the resource because of the movement of the Earth around the sun. Reference data for solar radiation, which are the solar constant and spectral distribution, are established for the average sun–earth distance conditions. The solar constant is the energy that falls per unit area and per unit time on a surface normally oriented to the direction of the propagation of solar radiation and located outside the Earth’s atmosphere. This value varies slightly and is equivalent on average to 1367 W m^−2^ [2,3]. The radiation emitted by the sun is distributed over a wide spectrum of wavelengths, with most of the radiated energy corresponding to the portion between 150 and 3000 nm with a spectral distribution very similar to that produced by a black body at 5777 K. About half of this energy falls within the visible band, 390 to 770 nm. The rest almost entirely corresponds to infrared radiation, with a small percentage of ultraviolet radiation.

The plant microbial fuel cell (PMFC) uses plants and bacterial communities, principally the *Geobacter genus*, to generate electricity through natural processes that occur in the roots of plants [4,5,6,7,8,9,10,11]. One of the main challenges with the current state of technology is its power output. Even though theoretical power output is estimated at 3.2 W m^−2^ in systems with plants as the sole organic matter source [12,13,14], the power yield of a PMFC can reach 1000 GJ ha^−1^ year^−1^, according estimations of a multidisciplinary European research consortium and that a hypothetical PMFC power yield of 21 GJ ha^−1^ year^−1^ is generally associated to a traditional device [12]. On the other hand, when the energy creation is applied in an indigenous habitat, the power yield of a PMFC is assessed to be limited to 1.6 MW km^−2^ [9], while wind turbines and solar panels could create 5 to 7.7 MW km^−2^ and 4.5 to 7.5 MW km^−2^, respectively, on a typical windy day under Western European conditions [9].

Plants produce organic material from sunlight and environmental CO_2_ through photosynthesis. About 70% of organic material ends up in the soil as dead root debris, mucilage, and exudates [15,16,17,18,19,20]. The organic material that fixes the plants in the soil can be oxidized by the rhizosphere bacterial community’s releases of CO_2_, protons (H^+^), and electrons (e^−^). These e^−^ are donated by bacteria while operating their metabolic processes. The H^+^ and dissolved O_2_ are part of O_2_ reduction reaction (ORR) mechanisms, generating electrical work during e^−^ reduction [18,21]. A PMFC device is considered a specific solar cell, transforming biochemical energy to electricity under specific electrochemical work [9]. The materials used as electrodes have total relevance. Various materials, such as Cu, Ni, C (graphite), Ti, among others, have been tested as anode materials because of their catalytic activity [22,23]. Cu metal has the following characteristics: (i) it is known to be an antimicrobial material (Cu ions are toxic for planktonic bacterial cells and prevent biofilm formation), (ii) Cu electrical conductivity is close to 900 times greater than polycrystalline graphite, (iii) it decreases internal resistance, and (iv) it considerably reduces the amount of required electrode material [24,25,26,27]. Cu corrosion products avert the creation of electrochemically active biofilms over the Cu anode electrode, especially during the start-up of the electrochemical period when biofilm formation still must take place, and anode potential due to the missing reduction power of a fully established microbial biofilm may shift into corrosion potential [28,29]. On the other hand, stainless steel AISI 316L materials are used as cathode electrodes with an interesting performance [20,30]. The potential difference between the anode and cathode simplifies the movement of e^−^ by an external circuit to decrease an e^−^ acceptor such as O_2_ at the cathode surface [31,32]. Using this renewable energy is imperatively necessary for the development of agriculture, even in the most desert-like climates with locally grown plants.

This work analyzes the in situ behavior of CAM plants for potential use as a PMFC. The study is based on extensive data obtained from the application of AISI 316 and Cu electrodes in different CAM plants and assesses their growth under the following parameters: (i) high levels of solar radiation, (ii) tolerance to water stress, (ii) resistance to temperature variations present, and (iv) different open circuit potential (OCP) behavior.

## 2. Results and Discussion

**Figure 1:**
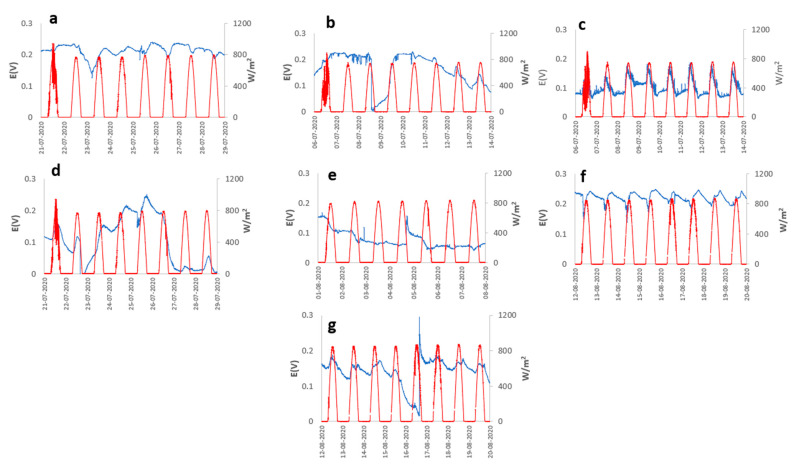
Potential (blue line) and Solar radiation (red line) vs. time for (**a**) *Austrocylindropuntia subulata*, (**b**) *Aloe perfoliata*, (**c**) *Cereus jamacaru*, (**d**) *Malephora crocea*, (**e**) *Agave potatorum*, (**f**) *Kalanchoe daigremontiana*, and (**g**) *Aloe arborescens*.

**Figure 2:**
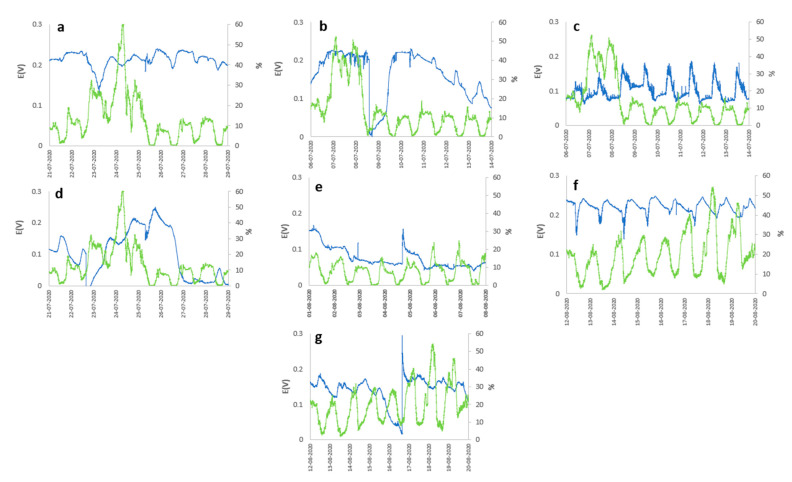
Potential (blue line) and Relative Humidity (green line) vs. time for (**a**) *Austrocylindropuntia subulata*, (**b**) *Aloe perfoliata*, (**c**) *Cereus jamacaru*, (**d**) *Malephora croce*a, (**e**) *Agave potatorum*, (**f**) *Kalanchoe daigremontiana*, and (**g**) *Aloe arborescens*.

**Figure 3:**
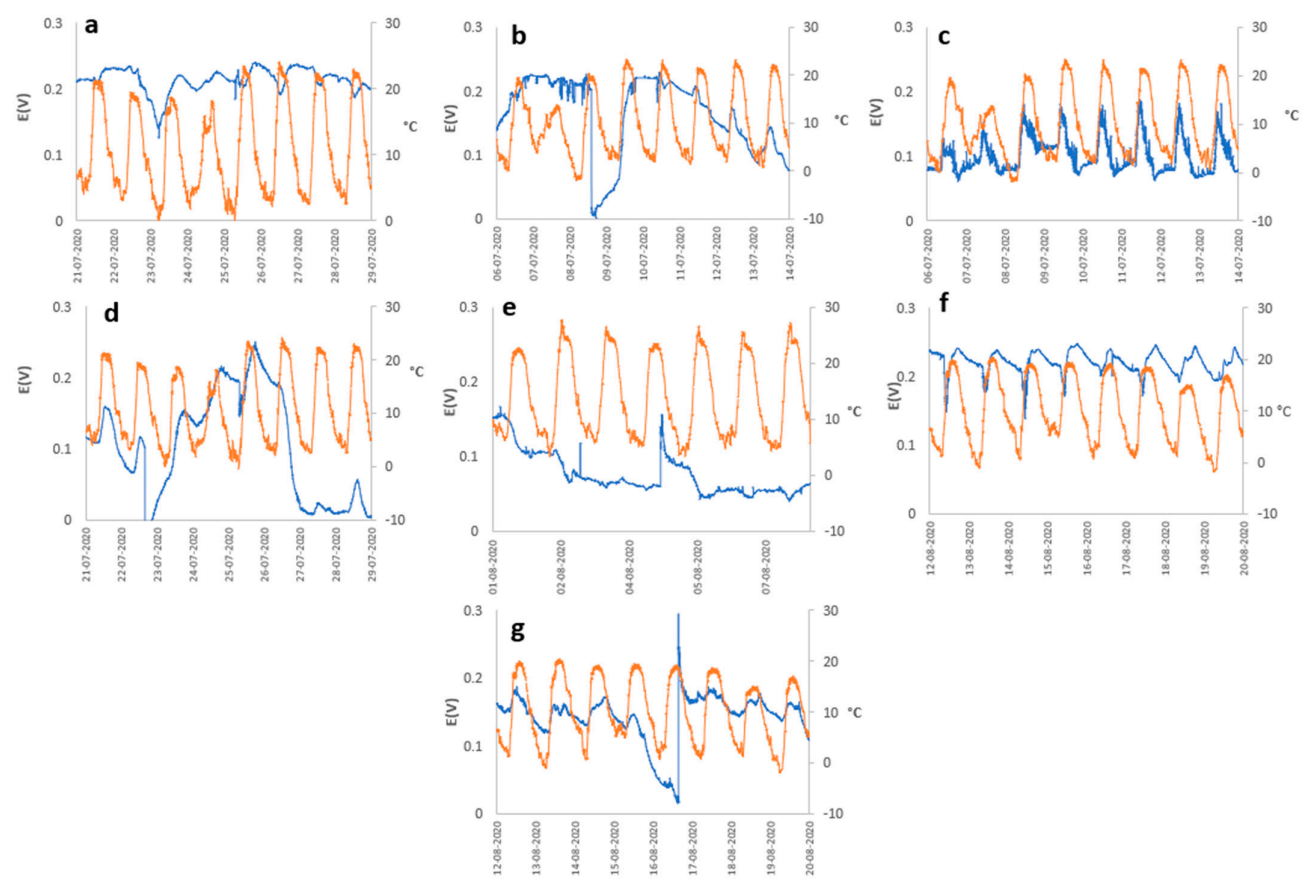
Potential (blue line) and Ambient Temperature (orange line) vs. time for (**a**) *Austrocylindropuntia subulata*, (**b**) *Aloe perfoliata*, (**c**) *Cereus jamacaru*, (**d**) *Malephora crocea*, (**e**) *Agave potatorum*, (**f**) *Kalanchoe daigremontiana*, and (**g**) *Aloe arborescens*.

The behavior of the open circuit potential (OCP) of different PMFCs concerning meteorological variables, such as (i) solar radiation, (ii) relative humidity, and (iii) ambient temperature during a specific period are represented in Figure 1, Figure 2 and Figure 3. Generally, the OCP performance of each PMFC presents notable variability; however, they all present cyclical behavior concerning the atmospheric variables analyzed in this study.

The species *Cereus jamacaru* and *Kalanchoe daigremontiana* represented in Figure 1c and Figure 1f, respectively, showed notable cyclic behavior of the OCP concerning solar radiation, generating an OCP of 0.187 and 0.248 V with a radiation level of 886.5 W m^−2^ and relative humidity in 8 days of analysis. The same cyclic behavior, despite not being as evident, was exhibited by *Austrocylindropuntia subulata* represented in Figure 1a, but from the second day of measurement, presented its maximum OCP of about 0.240 V in the periods of maximum solar radiation and relative humidity of about 942 W m^−2^ and 61% represented in Figure 2g. On the other hand, *Aloe arborescens* and *Aloe perfolia* represented in Figure 1g and Figure 2b, respectively, showed slight peaks in periods of higher solar radiation, generating a maximum OCP of 0.295 V with solar radiation and an relative humidity of 872 W m^−2^ and 54%. However, the temporal variation in the potential for the *Aloe arborescens* were more stable compared to *Aloe perfolia* (Figure 1b) and other species. Contrary to the previous cases, *Malephora crocea* and *Agave potatorum* represented in Figure 2d and Figure 2e, respectively, did not show a good performance, reaching maximum OCP values of 0.250 and 0.167 V, respectively.

The behavior of PMFCs with regard to the ambient temperatures, as is shown in Figure 3, was generally maintained at a relationship that was inversely proportional to humidity and directly proportional to solar radiation. Consequently, in most cases, the results resembled those obtained from the analysis of solar radiation and relative humidity. The performance of each of the seven plant species, although not all of them behaved in the same way, was an expected result since they were different plant species with the same photosynthetic pathway. This variability in performance can be attributed to the different rates of organic matter fixation through exudation in the rhizosphere, which in turn led to variations in the colonies of electrogenic microorganisms.

Meteorological variables needed to play a fundamental role in the performance of the PMFC, as was observed in the results; in general, the majority of the plant species presented cyclical behavior for the generation of electrical energy, being directly related to the cycle of solar radiation from which two assumptions can be deduced—the first assumption is that the performance was influenced by increased photosynthetic activity driven by heightened stomatal activity, leading to a greater secretion of organic matter into the rhizodeposits. This, in turn, stimulated greater activity of the rhizobacteria, responsible for generating electrical energy, due to increased electrical interactions at the cell membrane, as they had a greater amount of resources for metabolism. The second assumption regarding the correlation of cell potential with meteorological variables can be linked to the availability of molecular O_2_ in the electrochemical cell. O_2_ availability increased during periods of higher solar radiation, which, in turn, lead to an increase in ambient temperature (see Figure 3). The elevated temperature promoted greater evaporation of water from the substrate, thereby reducing humidity. This reduction in humidity may be associated with the potential contribution of moisture to the substrate. This effect would trigger an increase in efficiency in the anodic zone, since the greater the availability of O_2_, the greater the rate of reduction of O_2_ in H_2_O due to the effect of the ORR, with the 4e^−^ consume predominating the direct formation of H_2_O, compared with the 2e^−^ consume, which is less efficient and generates H_2_O_2_, which is harmful to microorganisms and would affect the generation of electrical energy by reducing the microbial population of the electrochemical system.

**Figure 4:**
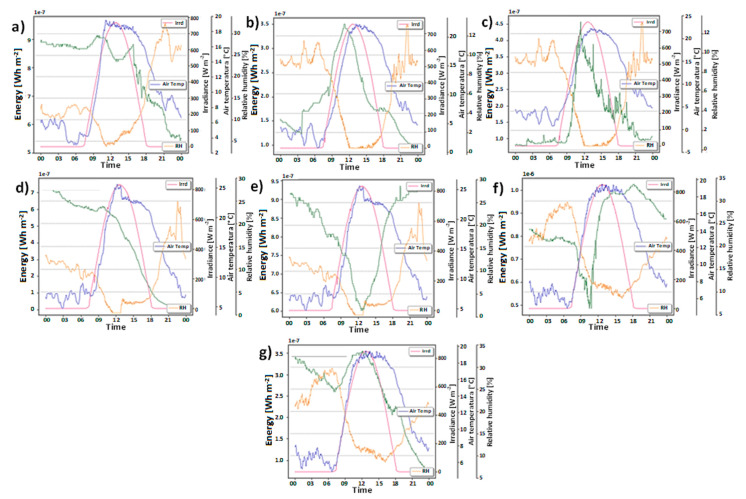
Energy generation performance per day in Wh m^−2^ of electrode; (**a**) *Austrocylindropuntia subulata*, (**b**) *Aloe perfoliata*, (**c**) *Cereus jamacaru*, (**d**) *Malephora crocea*, (**e**) *Agave potatorum*, (**f**) *Kalanchoe daigremontiana*, and (**g**) *Aloe arborescens*.

Figure 4 illustrates the energy recovery performance of each plant concerning prevailing environmental parameters, including solar radiation, relative humidity, and temperature according to Equations (8)–(10) (see Section 3.4). These graphs visually represent the correlation between these factors and the amount of energy harnessed by the plants through their microbial fuel cell systems. By examining the trends depicted in Figure 4, we can gain valuable insights into how variations in solar radiation, relative humidity, and temperature influence the overall energy generation potential of plant microbial fuel cells. The data presented in this figure play a crucial role in understanding and optimizing the efficiency of these sustainable energy conversion systems in diverse environmental conditions.

The experiment using CAM plants in PMFCs yielded promising results, showcasing the energy recovery capabilities of each plant. The accumulated energy recovery values, measured in mWh m^−2^ of electrodes, are as follows: (i) *Aloe perfoliata* demonstrated an impressive energy generation of 5.9 mWh m^−2^, (ii) *Cereus jamacaru* exhibited a respectable energy production of 2 mWh m^−2^, (iii) *Austrocylindropuntia subulata* displayed a remarkable energy generation of 8.8 mWh m^−2^, (iv) *Agave potatorum* demonstrated a noteworthy energy production of 7.8 mWh m^−2^, (v) *Malephora crocea* showcased a considerable energy generation of 3.2 mWh m^−2^, (vi) *Kalanchoe daigremontiana* displayed a significant energy production of 9.4 mWh m^−2^, (vii) *Aloe arborescens* exhibited a commendable energy generation of 4.1 mWh m^−2^. These energy recovery values demonstrate the potential of various CAM plants as efficient candidates for sustainable energy conversion in PMFC systems. The results offer valuable insights for optimizing and selecting suitable plant species to maximize energy generation in different environmental conditions, paving the way for eco-friendly and renewable energy solutions.

Plants have a characteristic that allows them to be grouped into three different types, depending on the environment in which they develop. They will have, to a greater or lesser extent, regulated something known as photorespiration, which is responsible for regulating the loss of water present in the plants. The photosynthetic pathways can be classified into three classes: C3, C4, and CAM [33]. The plants in each class differ from each other. The efficiency of C4 plants in the photosynthesis process is higher than the other categories [19], reaching a maximum limit of 6% compared to C3 and 4.6% compared to CAM plants. They produce a photosynthetic efficiency that defines the rate of the conversion of solar energy into organic materials, thus allowing for a greater proliferation of microorganisms. Understanding the photosynthetic pathways of plants is essential for choosing the right plant for the system. On the other hand, CAM plants that inhabit arid regions differ from C3 and C4 due to their ability to absorb CO_2_ at night, leading to water conservation in their tissues. CAM plants grow very slowly, resulting in lower biomass production than C3 and C4 plants [34]. Therefore, the choice of the type of plant would be even more relevant if it is to be used in places such as the north of Chile, where radiation levels are very high, averaging around 1000–1300 W m^−2^ which is highlighted in violet. These areas represent some of the highest rates of solar radiation in Chilean territory. CAM plants are adapted to dry and arid climates. They use the Crassulacean acid metabolism (CAM) pathway to minimize photorespiration. One characteristic of this metabolic pathway is that instead of separating light-dependent reactions and the utilization of CO_2_ in the Calvin cycle, CAM plants temporally separate these processes (CO_2_ fixation). This means that during the night, these plants open their stomata, allowing CO_2_ to diffuse through the leaves, and consequently, CO_2_ is fixed in the oxaloacetate by PEP carboxylase, which is the same step in the metabolism of C4 and is subsequently converted into malate or another organic acid. This acid is later stored in the vacuoles until the next day. The result of this process allows CAM plants to carry out photosynthesis during the day without the need for opening their stomata. This product has organic acids stored in its vacuoles that decompose to release CO_2_ and enter it into the Calvin cycle. Having controlled the release of CO_2_ results in maintaining a high concentration of it around the rubisco [15,21,33,34].

During the cathodic sub-process, the main e^−^ acceptor is dissolved O_2_, which functions as a reductant and, in the presence of a suitable catalyst, can form H_2_O. The process of e^−^ acceptance and subsequent O_2_ reduction is known as the oxygen reduction reaction (ORR) mechanism. This process can occur via the 4e^−^ transfer process or via a 2e^−^ transfer process, which largely depends on factors such as the catalyst (cathode material) and the pH that define the reaction path. Generally, there are two different pathways for the reaction of O_2_ molecules; the first is the 4e^−^ pathway, where O_2_ is reduced directly to two H_2_O molecules in an acid medium or into four OH^−^ molecules in a basic medium [7,35] as seen in the following reactions:

(1) $$O_{2}+4H^{+}+4e^{-}\to 2H_{2}O\left(Acid\;media\right)$$

(2) $$O_{2}+2H_{2}O+4e^{-}\to 4OH^{-}\left(Alkaline\;or\;neutral\;media\right)$$

The second alternative for the reduction of the O_2_ molecule is through the path of two e^−^; this is considered an incomplete process that occurs in two steps, where O_2_ is reduced to H_2_O_2_ in acid soil in the case of the reduction of the O_2_ molecule in alkaline medium being reduced to OH^−^, as shown below [35].

(3) $$O_{2}+2H^{+}+2e^{-}\to H_{2}O_{2}\left(Acid\;media\right)$$

(4) $$2H^{+}+2e^{-}+H_{2}O_{2}\to 2H_{2}O\left(Acid\;media\right)$$

(5) $$O_{2}+H_{2}O+2e^{-}\to OOH^{-}+OH^{-}\left(Alkaline\;media\right)$$

(6) $$OOH^{-}+H_{2}O+2e^{-}\to 3OH^{-}\left(Alkaline\;media\right)$$

**Figure 5:**
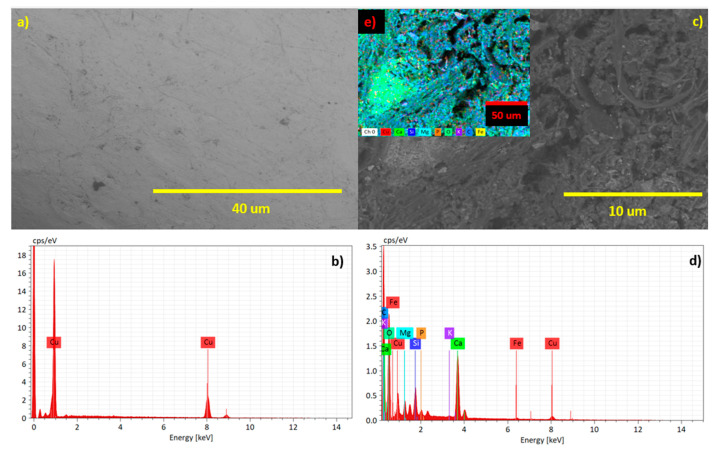
Surface morphology of the Cu anode electrode used for bioenergy recovery, (**a**) Cu electrode before bioenergy recovery, (**c**–**e**) Cu electrode after bioenergy recovery, (**b**) EDX elemental analysis of the Cu electrode before bioenergy recovery, and (**d**) EDX elemental analysis of the Cu electrode after bioenergy recovery.

The plant’s main function is to provide nutrients to bacteria through photosynthetic reactions and generate an ideal space for the proliferation of bacterial colonies [36]. An unhealthy plant will have a lower yield, which means that it will run out of nutrients to feed bacterial colonies present in the rhizosphere [37]. The PMFC (Figure 5) is based on the bioelectrochemical activity of microorganisms with the ability to degrade organic material and nutrients into simpler molecules such as CO_2_ (metabolic waste) and also release e^−^ and H^+^; these processes occur in areas with high microbial activity and ideally low levels of O_2_, because the electrogenic bacteria (e^−^ donors) are mostly anaerobic and/or facultative bacteria. For this reason, the anodic zone (where the anodic electrode is located) is where the processes occur in their entirety. The capture and transfer of electrons that occur when bacterial colonies form a biofilm on the anode surface, becoming electron donors, is known as the process of extracellular electron transfer [38].

The anode in contact with the ground is populated with a community of electrogenic bacteria concentrated on its surface. The bacteria community releases e^−^ during the metabolic process of nutrients present in the substrate, creating a current circuit during the reduction process over the cathode surface. The reaction involved during the anodic sub-process is the following [39,40]:

(7) $${CH}_{3}{COO}^{-}+4H_{2}O\to 2{HCO}_{3}^{-}+{9H}^{+}+{8e}^{-}$$

Figure 5 shows the anode electrode’s morphological change during the electrochemical process before and after the bioprocess and contact with this bacteria colony. Figure 5a indicates the electrode is pure Cu before bioenergy recovery, and Figure 5c,e shows the result of the anode after bioenergy recovery, in where the dissolution of Cu is present as Cu → Cu^2+^ + 2e^−^, and the presence of other elements such as Ca, Mg, C, K, and P and trace Fe, are due to the adhesion of nutrients on the Cu surface.

Natural electron acceptors are oxygen, nitrogen, and sulfur, among others [9]. Great influencing factors on the efficiency and electricity production of the plant are climatic conditions, salinity of the soil, relative humidity, sun exposure time, radiation, and the type of bacteria predominantly found in the root’s piliferous zone.

The e^−^ transfer process occurs from proteins in the outer membrane via intracellular e^−^ carriers such as NAD^+^/NADH. This mechanism grants certain bacteria the capability to participate in the electrogenic process. The interaction that occurs between the e^−^ donor and the electrode is known as an extracellular electron transfer, and it occurs in three steps: (i) microbial oxidation (metabolic process), (ii) transfer from the intracellular carrier, and (iii) extracellular electron transfer. In the area near the anode, oxidation reactions occur due to microbial activity [41]. Microorganisms can oxidize e^−^ donors through extracellular electron transfers. Therefore, this process involves both the internal and external e^−^ transport of the cell, of which three transfer mechanisms are: (i) a direct e^−^ transfer involving enzyme complexes associated or attached as a biofilm to the anode, (ii) an indirect e^−^ transfer, when a soluble organic or inorganic compound is oxidized in the cell and subsequently diffuses toward the acceptor (anode electrode), and (iii) a direct transfer of e^−^ through a conductive matrix known as pili or nanowires.

## 3. Materials and Methods

### 3.1. Open Circuit Potential (OCP) Monitoring

The experimental procedure was designed to examine the potential (E) generation from in situ CAM plants in Calama located in the Atacama Desert. The electrodes used were a Cu plate as an anode and a stainless steel AISI 316L plate as a cathode of 10 cm^2^. A PMFC single chamber with electrodes methodically positioned around the rhizosphere (1 mm distance) represented a sophisticated bioelectrochemical marvel. It ingeniously tapped into the dynamic metabolic interplay of soil-dwelling microorganisms amidst plant roots to orchestrate a remarkable feat: electricity generation. This ingenious setup capitalized on the intrinsic synergy between plant biology, microbial consortia, and conductive materials to efficiently transfer electrons, culminating in the electric current generation. The rhizosphere, which is a bustling realm where plant roots and the soil meet, became a nexus of activity, with roots acting as a means of propagation for resident microorganisms located in this zone. These electrons, as tiny messengers of potential energy, traversed the conductive material of the anode electrodes, forging an intimate connection between the living world below and the inanimate electrode realm above. This study was designed considering two study stations in the Atacama Desert climate. The CAM species were selected for their characteristics such as resistance to high levels of solar radiation, tolerance to water stress, and resistance to sudden variations in temperature. All the experiments used a data logger AEMC model L452 for the continuous collection of the potential fluctuations related to the OCP monitoring. The PMFC was monitored for periods of 8 days on average. The recording intervals of sampled information are every 1 s for the variables potential, temperature, relative humidity, and radiation using the horizontal global radiation.

### 3.2. Electrodes Surface Characterization

The morphological analysis of the cathode and anode electrode surface was characterized by scanning electron microscopy (SEM) using a Hitachi SU 500 microscope.

### 3.3. Meteorology Monitoring

**Figure 6:**
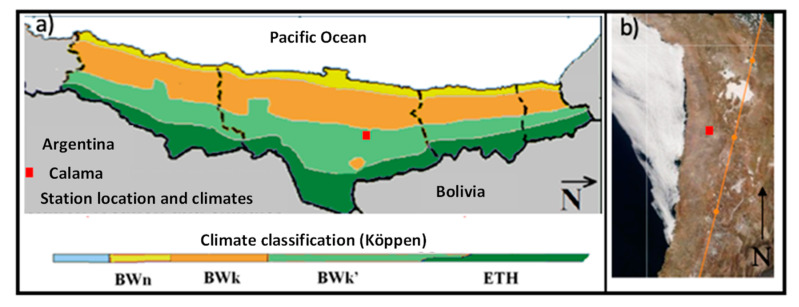
(**a**) The location of Calama and climatic classification according to Köppen, Antofagasta Region, Chile. (Image based on Marzo et al.) [44], (**b**) This picture was taken by a MODIS-Terra instrument from space, on 23 August 2022 (http://aeronet.gsfc.nasa.gov). The red square highlights the location of Calama.

The experiment was carried out in Calama (Figure 6). This city is located at 2260 m.a.s.l. in the Atacama Desert (22°28′0″ S, 68°55′60″ W). It presents a Köppen climate rating of type BWk’, which corresponds to a cold arid zone (Figure 6a) [42]. It offers an excellent quality of solar resources, which shows an average of 7.22 kWh m^−2^ of global horizontal irradiance (GHI) per day, obtaining a total of 2632 kWh m^−2^ annually. This zone is characterized by annual precipitation that does not exceed 50 mm year^−1^, which is reflected in its clear skies throughout the year as shown in Figure 6b (the satellite image was taken by MODIS-Terra, an instrument from space, 23 August 2022) [43]. The atmospheric parameters involved in this study were evaluated with a meteorological station in the city. Ambient Temperature (Tamb) and relative Humidity (RH) were measured with a young thermometer and hygrometer models CS215-L11 and hmp60-L11, respectively. Global horizontal irradiance (GHI) was measured with a Kipp & Zonen CMP21 pyranometer. The resolution of each measured parameter was 1 s and was recorded as an average value per minute.

### 3.4. Experimental Data Analysis

The model L452 datalogger records data by potential with a voltage range of 0 to 1 V and an internal impedance of 1 MΩ (ref). The experimental data for each test were conducted between 6 July 2020 and 19 August 2020 with a sampling resolution of 1 min. The cumulative energy generated by the plants needs the following sequence:

(a) The current generated during the experiments was calculated using Ohm’s law, according to the following expressio
  (8) $$I_{p}=\frac{V_{p}}{R}$$
  where $I_{p}$ is the current generated from the plants, $V_{p}$ is the potential generated for the plants of the experimental measurements, and R is the impedance resistance of the equipment, respectively.
(b) The power generated by the plants is calculated according to Equation (9)
  (9) $$P_{p}=V_{p}\cdot I_{p}$$
  where $P_{p}$ is the power generated from the plants.
(c) Finally, accumulated energy recovery from the plant was obtained by calculating the area under the curve using the trapezoid method according to the following expression:
  (10) $$E=\frac{\left(P_{p\_n}+P_{p\_n+1})\cdot (t_{n+1}-t_{n}\right)}{2}$$
  where E is the energy generated for each plant during the experiment, n is the number of samples, $P_{p\_n}$ is the power calculated using Equation (9) for the n sample, and t is the time [45,46].

### 3.5. CAM Species Plants as PMFC

The species used were seven and are (i) *Aloe perfoliate* [47,48], (ii) *Cereus jamacaru* [49,50,51,52,53], (iii) *Austrocylindropuntia subulate* [54,55,56], (iv) *Agave potatorum* [57,58,59,60,61], (v) *Malephora crocea* [33,62], (vi) *Kalanchoe daigremontiana* [63,64,65,66,67,68], and (vii) *Aloe arborescens* [69,70,71]. The soil used was a commercial composed of forest residues such as pine bark and sawdust and seaweed residues, with an apparent density of 0.4–0.7, pH (Dilution 1:5) of 5–8.5, Electrical Conductivity (EC) (Dilution 1:5) < 3 dS/m, and C/N relation < 50 for each PMFC. Climate condition monitoring was not controlled, and it was subjected to extreme levels of solar radiation, high temperatures, and fluctuations in the relative humidity. The water supply to the system was controlled by watering the plants with only 100 mL every 7 seven days (before installing the electrodes) to generate a medium with high water stress.

## 4. Conclusions

Based on the analysis conducted, CAM plants emerge as a promising and sustainable alternative for utilization in PMFCs, particularly in regions characterized by high solar radiation and arid or hyper-arid climates such as the Atacama Desert in Northern Chile. The performance of PMFC-employing CAM plants underscores the significance of nutrient availability, specifically Ca, Mg, C, K, and P, as well as trace elements like Fe and Cu in optimizing electricity generation efficiency.

Among the CAM plants studied, *Kalanchoe daigremontiana* exhibits exceptional performance metrics. It demonstrates the highest electrical energy generation, remarkable consistency in cell potential, and minimal variations. This plant achieves a maximum cell potential of 0.248 V and a minimum of 0.139 V, with a recurrence rate of approximately 89% within the voltage range of 0.200 to 0.250 V. These results position *Kalanchoe daigremontiana* as the first candidate for further exploration on a larger scale due to its noteworthy attributes, including low variability in the open circuit potential values. This low variability corresponds to a remarkable energy production of about 9.4 mWh m^−2^ of the electrode, further highlighting its viability for practical implementation. The energy estimate of energy generated by each CAM plant studied can be considered unique information which contributes to the scientific development of plant species adapted to desert climatic zones. However, while the potentials obtained from the metabolic processes of various CAM plant species exhibit relatively minor differences under the given conditions, determining the ultimate optimal species remains a challenge. Consequently, it becomes imperative to shift the focus of research toward the design and engineering of the electrochemical system itself. This approach aims to enhance energy recovery, thereby accommodating the extreme environmental conditions characteristic of the Atacama Desert, where this study was conducted.

Proposed avenues for further investigation involve the integration of soilless cultivation technologies within the context of the electrochemical system. This strategic approach is intended to provide insights into how CAM plants interact with the electrochemical environment when grown in controlled conditions, potentially yielding significant improvements in performance and energy output. In the context of the nutrient requirements for bacterial activity in the rhizosphere of these CAM plants within PMFC, it is important to acknowledge that the specific needs of bacteria can vary based on the particular bacterial species, chosen plant species, and the composition of the soil. Furthermore, the availability of nutrients, including Ca, Mg, C, K, P, Fe, and Cu, is intrinsically linked to parameters such as pH, temperature, and humidity. These factors collectively influence microbial metabolic processes and subsequent electricity generation in the PMFC systems. CAM plants, particularly exemplified by *Kalanchoe daigremontiana*, hold great promise as a sustainable solution for PMFC electrochemical systems in challenging environmental conditions. This study underscores the critical role of nutrients and trace elements in optimizing PMFC performance. Moving forward, a shift towards system-level design and engineering, coupled with controlled cultivation techniques, will be crucial for advancing the practical application of this innovative technology in extreme environments.

## Data Availability

The processed data are available from the corresponding author.
